# The impact of haemostatic agent used during robot‐assisted radical prostatectomy on post‐op infection and anastomotic leak

**DOI:** 10.1002/bco2.70023

**Published:** 2025-07-02

**Authors:** Jamie Krishnan, Rory Brennan, Osman El‐Koubani, Sailantra Sivathasan, Kevin Gallagher, Linda Taylor, Abhishek Sharma, Daniel Good, Alan McNeill

**Affiliations:** ^1^ Western General Hospital Edinburgh UK

**Keywords:** RARP, prostatectomy, haemostatic agent, cystogram, anastamotic leak, UTI

## Abstract

**Objective:**

Despite the widespread use of robotic‐assisted radical prostatectomy (RARP), considerable variation exists in practice with regard to postoperative cystograms prior to trial without catheter (TWOC).

Our practice was to undertake a cystogram at 7–14‐day post‐op and proceed with TWOC if the leak was small or absent.

In this study, we evaluate the risk factors for anastomotic leak and post‐op urinary tract infection (UTI), while assessing the impact of using powdered haemostat Arista AH on post‐op UTIs and cystographic leaks to rationalise the use of cystograms.

**Methods:**

The study was carried out in two phases, the first a retrospective review of 154 patients undergoing RARP between January and October 2022, where Oxitamp (an absorbable haemostat of oxidised regenerated cellulose) was used as the haemostatic agent.

The second phase was prospective, involving 62 patients between November and June 2023, in whom the powdered haemostat Arista AH (microporous polysaccharide haemospheres) was used instead.

Data were collated from a prospectively collected database (REDCap^Ò^) and electronic patient records. RARP was performed by two experienced surgeons using similar techniques. Analysis was carried out on R‐studios, using Fisher's exact test for categorical variables and unpaired student *t*‐test for continuous variables.

**Results:**

Post‐op UTI occurred in 19 (12%) patients in Cohort 1 (with Oxitamp) and leaks were associated with 15 (79%). In comparison, seven (11%) patients in Cohort 2 (with Arista AH) suffered from post‐op UTI, and only one (14%) was associated with leak.

There was an overall reduction in leaks on post‐op cystograms from 44 (29%) in Cohort 1 to only nine (15%) in Cohort 2 (*p* = 0.036).

One hundred per cent of the leaks in Cohort 2 were small, compared to nine (20%) in Cohort 1 (*p* = 0.0002).

**Conclusions:**

A selective approach can be implemented with regard to post‐op cystograms.

We propose that when using Arista AH cystograms should be carried out in patients in need of bladder neck reconstruction. We will also continue to undertake routine cystograms in those with a history of transurethral resection of prostate (TURP) or in salvage prostatectomy cases.

## 1 INTRODUCTION

Prostate cancer is one of the most common cancers in men, with one in eight being diagnosed within their lifetime.^1^ Robotic‐assisted radical prostatectomy (RARP) continues to be the most common surgical approach in the United Kingdom, with over 8000 procedures per year.^2^

Despite the widespread use of RARP, there is considerable variation in practice with regard to whether or not haemostatic agents are used and whether or not a postoperative cystogram is performed to assess for anastomotic leaks prior to a trial without catheter (TWOC). Guru et al. suggested a TWOC 8–10‐day post‐RARP without performing a cystogram was safe; however, other studies have supported performing a cystogram in selected cases.^3^, ^4^, ^5^

Furthermore, when cystograms are carried out, the postoperative timing is also variable.^6^, ^7^

A number of studies have identified risk factors for anastomotic leaks in order to promote increased selectivity in requesting cystograms rather than undertaking them in all patients routinely. Postoperative urinary tract infections (UTIs), prostate volume, difficult anastomosis and previous transurethral resection of prostate (TURP) have all been implicated as have factors such as console time, leak on intra‐operative stress test and patient body mass index (BMI).^5^, ^8^, ^9^

We sought to further evaluate the risk factors for anastomotic leak and postoperative UTI while assessing the impact of changing the haemostatic agent used from Oxitamp gauze to Arista AH in order to rationalise our use of cystograms prior to the trial without catheter. Arista AH is a plant‐based, non‐immunogenic powdered haemostat consisting of microporous polysaccharide haemospheres.^10^ Gilbert et al. demonstrated a reduction in the rate of lymphocoele formation when using Arista AH.^11^

## 2 MATERIALS AND METHODS

RARP was performed in a transperitoneal retropubic approach by two experienced surgeons (AMcN and DG), both operating across two sites on either the DaVinci X or Xi systems. None of the patients had preoperative UTI's. Both surgeons completed the anastomosis using Stratafix after Rocco's posterior rhabdosphincter reconstruction by a continuous 3,0 or 4,0 monocryl in two layers. An intra‐operative stress test with 120 mL of saline was performed in all cases via a 18Ch Foley catheter with 10 mL in the balloon. Prior to the introduction of Arista AH as the haemostatic agent both surgeons routinely placed Oxitamp gauze on each side of the anastomosis, over the neurovascular bundles, prior to undertaking an anterior reconstruction of the endopelvic fascia. Both surgeons maintained the same operative technique over the period of this study.

Cystograms prior to TWOC were routinely carried out in all cases and reported by an experienced uro‐radiologist with leaks classified as follows:

- Small: Leakage confined directly around the anastomosis;
- Medium: Extravasation extending to more distant areas around the anastomosis;
- Large: Large volume leakage extending even further from the anastomosis with potential complications.

**FIGURE A1:**
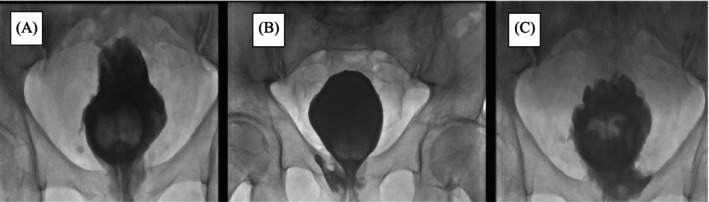
Cystograms demonstrating (a) small, (b) medium and (c) large leaks.

Figure A1 illustrates such leaks. Cystograms were carried out to ensure that premature removal of the catheter was avoided and to minimise the risk of anastomotic breakdown. Most cystograms were carried out between 7‐ and 14‐day postoperatively, depending on slot availability, the surgeon's subjective perioperative impression of the quality of the anastomosis and patient factors that would impact the stability and healing of the anastomosis. For example, for patients with a high BMI or narrow pelvis (making it technically challenging) or those who underwent previous radiotherapy, the surgeon may elect to perform the cystogram around the 14‐day mark.

Saline diluted iodinated contrast medium was instilled via the urethral catheter and antero‐posterior (AP) and oblique views were obtained in order to ascertain the integrity of the vesico‐urethral anastomosis. Leaks were graded as small, medium or large. Our practice is to undertake a TWOC following a cystogram if there is no leak or only a small leak. If there is a medium or large leak, then an additional cystogram is performed 7–14 days after the initial cystogram. This process was repeated until the leak was categorised as very small or there is no leak at which point a TWOC is then undertaken.

This study was carried out in two phases in a similar fashion to that reported by Gnanapragasam et al.^8^ The first was a retrospective review of 154 patients who underwent RARP between January and December 2022, prior to the introduction of Arista AH (Cohort 1). Oxitamp was used as the haemostatic agent in this cohort. The second phase was carried out prospectively and involved a cohort of 62 patients in whom Arista AH was used between June 2023 and October 2023 (Cohort 2). The study will analyse the impact changing the haemostatic agent has on cystographic leak rates; as Arista AH was anecdotally noted to yield favourable results with regard to leaks when the authors attended another centre where Arista AH was being used routinely.

Preoperative data collected included patient age, Charlson comorbidity index, BMI, presenting PSA, previous TURP or radiotherapy. Operative notes were reviewed to record the approach taken (transperitoneal vs. extra‐peritoneal), nerve sparing laterality, degree of bladder neck reconstruction and operative time. Postoperative data included Gleason score, tumour stage, gland size, margin status, timing of cystogram, presence and size of leak and post‐op UTI. The data were collected in a prospectively collected database (REDCap^Ò^) and electronic patient records.

Analysis was carried out on the software RStudios, by using Fisher's exact test for categorical variables and using unpaired student *t*‐test for continuous variables. Fisher's exact test was used rather than chi‐square test due to the small number of leaks when comparing across subgroupings in variables.

## 3 RESULTS

### 3.1 Patient demographics

Table 1 demonstrates the distribution of patient age, ASA scores, Charlson comorbidity index, pre‐op PSA and previous interventions across the two cohorts. No significant difference between the cohorts was noted with regard to patient demographics. All patients had a negative preoperative urinalysis at pre‐assessment to identify and treat potential urinary tract infections.

**TABLE 1:** Comparison of the number and size of leaks on cystogram between the two cohorts—Cohort 1 with Oxitamp as the haemostat and Cohort 2 with Arista AH.

	Cohort 1	Cohort 2	
Parameters	(*n* = 154)	(*n* = 62)	*p*‐Value
**Number of leaks on cystogram**	44 (29%)	9 (15%)	0.036
**Size of leak**			
Small	7 (16%)	9 (100%)	>0.001
Medium	17 (39%)	0 (0%)	
Large	15 (34%)	0 (0%)	
Missing	5 (11%)	0 (0%)	
**Mean days catheter in situ**			
All patients	20.47	13.05	>0.001
Leak	45.23	30.33	0.001
No leak	10.56	10.11	0.349

### 3.2 Leak rates

Post‐op cystograms demonstrated leaks in 44 (29%) patients in Cohort 1 compared to nine (15%) in Cohort 2. The overall reduction in leaks was statistically significant (*p* = 0.036). The leaks noted were classified as small, medium or large in seven (16%), 17 (39%) and 15 (34%), respectively, in Cohort 1, while all leaks were small in Cohort 2.

This was a significant reduction in the number of large or moderate leaks in Cohort 2 (*p* < 0.001). This is demonstrated in Table 2 below.

**TABLE 2:** Patient demographics between the two cohorts: Cohort 1 in which Arista AH was not used and Cohort 2 in which Arista AH was used.

	Cohort 1	Cohort 2
Parameters	(*n* = 154)	(*n* = 62)
**Age**		
Mean	67.23	67.01
Standard deviation	5.99	6.11
**BMI**		
Mean	28.71	28.25
Standard deviation	4.11	3.72
**Charlson index**		
Mean	2.62	2.7
Standard deviation	1.08	1.09
**Pre‐op PSA**		
Mean	11.39	9.21
Standard deviation	7.91	6.70
**Days from operation to cystogram**		
Mean	11.09	10.05
Standard deviation	5.27	2.43
**Previous radiotherapy**		
No previous radiotherapy	152 (99%)	61 (98%)
Previous radiotherapy	2 (1%)	1 (2%)
**Previous TURP**		
No previous TURP	150 (97%)	61 (98%)
Previous TURP	4 (3%)	1 (2%)
**ASA**		
1	32 (21%)	9 (15%)
2	106 (69%)	47 (76%)
3	16 (10%)	4 (7%)
Missing	0 (0%)	2 (3%)

Abbreviation: BMI, body mass index; TURP, transurethral resection of prostate.

### 3.3 Risk factors for leak

As shown in Table 3, analysis of Cohort 1 identified higher BMI (*p*‐value = 0.010) and ASA of 2 or greater (*p*‐value = 0.008) as risk factors for anastomotic leak. While the number of patients undergoing salvage prostatectomy was small, most cases had a leak however this sample was too small to perform Fisher's exact test. Age >70 years was associated with increased risk of anastomotic leak (*p*‐value 0.042).

**TABLE 3:** Risk factors identified in Cohort 1 and Cohort 2.

	Cohort 1			Cohort 2		
Risk factors	Leak (*n* = 44)	No leak (*n* = 110)	*p*‐Value	Leak (*n* = 9)	No leak (*n* = 53)	*p*‐Value
**Age**						
<70	23 (52%)	77 (70%)	0.042	6 (67%)	20 (38%)	0.148
≥70	21 (48%)	33 (30%)		3 (33%)	33 (62%)	
**ASA**						
<2	3 (7%)	29 (26%)	0.008	1 (11%)	8 (15%)	1.000
≥2	41 (93%)	81 (74%)		7 (78%)	44 (83%)	
Missing	0 (0%)	0 (0%)		1 (11%)	1 (2%)	
**BMI (mean)**	30.16	28.13	0.010	27.78	28.34	0.690
**Previous radiotherapy**						
No	42 (95%)	110 (100%)	0.0803	9 (100%)	52 (98%)	
Yes	2 (5%)	0 (0%)		0 (0%)	1 (2%)	1.000

Abbreviation: BMI, body mass index.

Conversely neither age, higher BMI nor ASA >2 were not shown to be a potential risk factor in the Cohort 2 (*p*‐value: 0.15, 1.0 and 0.690).

### 3.4 Intra‐operative and postoperative details

Table A1 highlights the impact of operative approach, operative times on cystographic leaks in Cohorts 1 and 2, respectively. The need for bladder neck reconstruction sutures and post‐op UTI were identified as risk factors for developing leaks in Cohort 1.

### 3.5 Post‐op UTIs

Symptomatic postoperative UTIs occurred in 19 (12%) patients in Cohort 1, and leaks were associated in 15 (79%) of these cases. Of the patients in Cohort 1 who did not have a post‐op UTI, leaks were noted in 29(21%) of cases (*p*‐value <0.001). In Cohort 2, only seven patients (11%), suffered from post‐op UTI's of which only one was associated with a leak. In addition, all the leaks in Cohort 2 were small in size.

### 3.6 Pathology

No significant difference was noted between the cohorts with regard to postoperative pathology as shown in Table A2.

Table 4 demonstrates that no individual pathological factors were associated with cystographic leaks in either cohort. Gland weight was not shown to have an effect on the likelihood of a leak for either Cohort 1 or 2.

**TABLE 4:** Impact of final pathology on cystographic leaks between cohorts.

	Cohort 1				Cohort 2			
Risk factors	All patients (*n* = 154)	Leak (*n* = 44)	No leak (*n* = 110)	*p*‐Value	All patients (*n* = 62)	Leak (*n* = 9)	No leak (*n* = 53)	*p*‐Value
**Mean gland weight**	59.49	59.22	60.24	0.850	59.02	59.48	56.78	0.751
**PT3**	67	24 (36%)	43 (64%)	0.071	31	2 (6%)	29 (91%)	0.147
**pN1**	9	5 (50%)	5 (50%)	0.097	1	0 (0%)	1 (100%)	1.000
**Positive margins**	35	24 (69%)	11 (31%)	0.002	13	1 (8%)	12 (92%)	0.418

## 4 DISCUSSION

This study demonstrates that the incidence of anastomotic leak on postoperative cystograms was reduced from 29% to 15% after changing haemostatic agent from Oxitamp gauze to Arista AH powder. This leak rate is in keeping with a number of published studies (Souto et al., Lepor et al.).^12^, ^13^ Not all such leaks are significant, and many patients, such as those with small leaks, can undergo safe TWOC.

Guru et al. reported that a TWOC on Days 8–10 without a cystogram is safe.^3^ They concluded that the cost and manpower needed for routine cystograms cannot be justified by the relatively low complication rate associated with leaks. Identifying which patients can safely avoid a cystogram prior to TWOC will help to reduce exposure to radiation, morbidity associated with the catheter and its implications on the patient's quality of life.^13^, ^14^, ^15^

Factors predictive of leaks, such as obesity, UTI, prostate size and previous transurethral surgery or radiotherapy, have been described in numerous previous studies. Although not all of these factors were found to be significant within this study, these still impact clinical decision making to some extent. Obesity, for example, can cause challenges with access and visibility leading to a suboptimal anastomosis potentially prone to leaks, while larger glands lead to less bladder neck preservation necessitating more reconstruction sutures. These points were also noted in studies by Tillier et al., Gnanapragasam et al. and Ramsden et al.^5^, ^8^, ^9^

The reduction in the leak rate noted, with the use of Arista AH instead of Oxitamp, from 29% to 15%, was statistically significant. There was a significant reduction in the number of moderate and large leaks, which is likely linked to the significant reduction in postoperative UTI seen between the cohorts studied.

Arista AH is typically absorbed within 24–48 h in comparison to Oxitamp, which takes 5–8 days and Surgicel at 7–14 days.^16^ We hypothesise that the faster absorption time of Arista AH contributes to the lower rates of postoperative UTI seen in our study, despite both Surgicel and Oxitamp having bactericidal properties. Infections in general are associated with poor healing and disrupting anastomotic healing in any context, so fewer infections translate to better conditions for healing postoperatively and thus fewer and smaller leaks on the cystogram.

The study is limited by being a single centre study with both surgeons applying the same operative techniques. Although it should be noted that this is also a positive in that there was no variation or change in approach between the cohorts aside from the choice of haemostat used.

We also appreciate that not all surgeons choose to routinely use a haemostatic agent following RARP, preferring a more selective approach. As a result, we acknowledge that this fact also acts to limit this study.

This study would also have benefited from a fully prospective and randomised approach, as in this study, only the second cohort, where Arista AH was used as the haemostat, was prospective. The first Oxitamp cohort was studied in a retrospective manner, which limits the conclusions that we can derive from this study.

## 5 CONCLUSION

On the basis of this study and previous data, it can be concluded that routine postoperative cystograms are not required in all patients, and a more selective approach can be implemented.^17^ We propose that when using Arista AH cystograms should only be carried out in patients >70 years of age and those in need of bladder neck reconstruction. We will also continue to undertake routine cystograms in those with a history of TURP, radiotherapy for prostate cancer.

## AUTHOR CONTRIBUTIONS

**Jamie Krishnan:** Write up. **Rory Brennan:** Statistics and analysis. **Osman El‐Koubani:** Data collection and literature review. **Sailantra Sivathasan:** Data collection and literature review. **Kevin Gallagher:** Data collection and paper edits. **Linda Taylor:** Paper edits. **Abhishek Sharma, Daniel Good and Alan McNeill:** Paper edits.

## CONFLICT OF INTEREST STATEMENT

The authors declare no conflicts of interest.

## Appendix

**TABLE A1:** Impact of intra‐op factors on cystographic leak.

	Cohort 1				Cohort 2			
Risk factors	All patients (*n* = 154)	Leak (*n* = 44)	No leak (*n* = 110)	*p*‐Value	All patients (*n* = 62)	Leak (*n* = 9)	No leak (*n* = 53)	*p*‐Value
**Surgical approach**								
Extra‐peritoneal approach	5	3 (60%)	2 (40%)		2	0 (0%)	2 (100%)	
Transperitoneal approach	149	40 (27%)	109 (73%)	0.133	49	9 (18%)	40 (82%)	1.000
**Never sparing**								
No nerve sparing	58	18 (31%)	40 (69%)	0.579	19	2 (11%)	17 (89%)	0.705
Unilateral nerve sparing	83	23 (28%)	60 (72%)	1.000	30	5 (17%)	25 (83%)	1.000
Bilateral nerve sparing	13	2 (15%)	11 (85%)	0.518	11	2 (18%)	9 (82%)	0.664
**Recon suture**								
No recon suture (bladder neck)	81	17 (21%)	64 (79%)	0.049	42	6 (14%)	36 (86%)	1.000
1 Recon suture (bladder neck)	20	8 (40%)	12 (60%)	0.608	2	0 (0%)	2 (100%)	1.000
2 Recon suture (bladder neck)	39	11 (28%)	28 (72%)	1.000	5	1 (20%)	4 (80%)	0.538
>2 Recon suture (bladder neck)	14	7 (50%)	7 (50%)	0.065	2	0 (0%)	2 (100%)	1.000
**Mean operating time (mins)**	133.32	139.25	130.99	0.234	129.31	122.5	130.5	0.563

**TABLE A2:** Comparing postoperative pathology between cohorts.

	Cohort 1	Cohort 2
Parameters	(*n* = 154)	(*n* = 62)
**ISUP grade groups**		
1	7 (5%)	3 (5%)
2	113 (73%)	44 (71%)
3	24 (16%)	10 (16%)
4	3 (2%)	3 (5%)
5	3 (2%)	0 (0%)
Missing	4 (4%)	2 (3%)
**Pathological T stage**		
pT2a‐c	86 (56%)	31 (50%)
pT3a	49 (32%)	28 (45%)
pT3b	18 (12%)	3 (5%)
Missing	1 (1%)	0 (0%)
**Never sparing**		
None	58 (38%)	19 (31%)
Unilateral	83 (54%)	30 (48%)
Bilateral	13 (8%)	11 (18%)
Missing	0 (0%)	2 (3%)
**Gland weight**		
Mean	59.49	59.02
Standard deviation	23.85	21.73
